# Exploring First Interactions Between Ostreid Herpesvirus 1 (OsHV-1) and Its Host, *Crassostrea gigas*: Effects of Specific Antiviral Antibodies and Dextran Sulfate

**DOI:** 10.3389/fmicb.2019.01128

**Published:** 2019-05-24

**Authors:** Claire Martenot, Nicole Faury, Benjamin Morga, Lionel Degremont, Jean-Baptiste Lamy, Maryline Houssin, Tristan Renault

**Affiliations:** ^1^Institut Français de Recherche pour l’Exploitation de la Mer, Laboratoire de Génétique et Pathologie des Mollusques Marins, La Tremblade, France; ^2^LABEO, Saint Contest, France; ^3^Département Ressources Biologiques et Environnement, Institut Français de Recherche pour l’Exploitation de la Mer, Nantes, France

**Keywords:** OsHV-1, antiviral antibodies, dextran sulfate, interaction, *Crassostrea gigas*, infection

## Abstract

Viral entry mechanisms of herpesviruses constitute a highly complex process which implicates several viral glycoproteins and different receptors on the host cell surfaces. This initial infection stage was currently undescribed for Ostreid herpes virus 1 (OsHV-1), a herpesvirus infecting bivalves including the Pacific oyster, *Crassostrea gigas*. To identify OsHV-1 glyproteins implicated in the attachment of the virus to oyster cells, three viral putative membrane proteins, encoded by ORF 25, 41, and 72, were selected and polyclonal antibodies against these targets were used to explore first interactions between the virus and host cells. In addition, effects of dextran sulfate, a negative charged sulfated polysaccharide, were investigated on OsHV-1 infection. Effects of antiviral antibodies and dextran sulfate were evaluated by combining viral DNA and RNA detection in spat (*in vivo* trials) and in oyster hemolymph (*in vitro* trials). Results showed that viral protein encoded by ORF 25 appeared to be involved in interaction between OsHV-1 and host cells even if other proteins are likely implicated, such as proteins encoded by ORF 72 and ORF 41. Dextran sulfate at 30 μg/mL significantly reduced the spat mortality rate in the experimental conditions. Taken together, these results contribute to better understanding the pathogenesis of the viral infection, especially during the first stage of OsHV-1 infection, and open the way toward new approaches to control OsHV-1 infection in confined facilities.

## Introduction

Ostreid herpesvirus 1 (OsHV-1) has been a major threat to Pacific cupped oyster cultivation in Europe over the last decades, especially for French oyster farmers. This virus—assigned to the *Herpesvirales* order and to the *Malacoherpesviridae* family—induced mortality of early life-stage oysters (Le Deuff and Renault, 1999; Davison et al., 2005, 2009). Mass mortality outbreaks among Pacific oysters, *Crassostrea gigas*, have been regularly reported throughout the world, and consequently dramatic losses in production were recorded, generating a decline in the oyster farming industry in several places. The conventional vaccination strategy appears inappropriate due to the lack of specific cell immunity in invertebrates. In addition, chemotherapy and immune stimulation are unsuitable to oyster cultivation in the open field, even if these approaches could be considered in closed environments such as hatcheries. Currently control measures based on restrictions of oyster movements by authorities, modification of cultural practices and resistance breeding constitute serious ways to limit virus propagation and to decrease mortality rates. For several years, OsHV-1 (GenBank accession no. AY509253, “reference” type) and variants had been detected during mortality outbreaks or without oyster mortality around the world, such as in France (Nicolas et al., 1992; Renault et al., 1994; Renault and Lipart, 1998; Le Deuff and Renault, 1999; Garcia et al., 2011; Martenot et al., 2011, 2015), Ireland (Lynch et al., 2012; Peeler et al., 2012; Clegg et al., 2014), Spain (Da Silva et al., 2008; Aranguren et al., 2012; Roque et al., 2012), Italy (Dundon et al., 2011; Domeneghetti et al., 2014), Portugal (Batista et al., 2014), South Korea (Hwang et al., 2013; Jee et al., 2013), United States (Friedman et al., 2005; Burge et al., 2006, 2011), Mexico (Grijalva-Chon et al., 2013), Australia (Jenkins et al., 2013; Paul-Pont et al., 2013a,b, 2014), Brazil (Mello et al., 2018), and New Zealand (Renault et al., 2012; Bingham et al., 2013; Keeling et al., 2014; Whittington et al., 2015). Since 2008, a variant called μVar has been mainly detected in French oyster samples and is characterized by 26 mutations in two regions of the viral genome: the C region (open reading frame (ORF) 4/5) and an IAP region (ORF 42/43) (Segarra et al., 2010; Martenot et al., 2011; Renault et al., 2012). Investigations performed over the last decades illustrated the efforts to better characterize OsHV-1 genetic diversity and its geographical distribution (Renault et al., 2012; Martenot et al., 2013, 2015; Burioli et al., 2016, 2017, 2018; Bai et al., 2017, 2019; Abbadi et al., 2018). Other studies focused on the OsHV-1 replication cycle by analyzing viral gene expression during an experimental OsHV-1 infection or *in vitro* approaches (Renault et al., 2011; Jouaux et al., 2013; Rosani et al., 2014; Segarra et al., 2014b; Morga et al., 2017). This knowledge was completed by recent results on tissue distribution of viral DNA, RNA and proteins (Schikorski et al., 2011a; Corbeil et al., 2015; Martenot et al., 2016; Segarra et al., 2016). Special interest is also given to host response to OsHV-1 infection by notably investigating relative expression of immune oyster genes (Renault et al., 2011; Jouaux et al., 2013; Rosani et al., 2014; Segarra et al., 2014a,b; Green et al., 2015; Morga et al., 2017; Green and Speck, 2018). The lack of bivalve cell lines complicates the understanding of the OsHV-1 infection mechanism and particularly interactions between the virus and its host cells. The present study attempts to better understand these interactions at protein level by approaches not already used in this model.

Polyclonal antibodies against three viral putative membrane proteins were incubated separately or in combination with OsHV-1 before injection of viral suspension in the adductor muscle of spat (*in vivo* trials) or before contact with hemolymph from adult oysters (*in vitro* trials). Viral membrane proteins play a major role in the earliest stage of infection during attachment and entry of the virus into host cells. The objective of incubating antiviral antibodies and OsHV-1 was to block potential viral proteins involved in molecular interactions between both partners and consequently to give new information about the first step of viral infection.

In addition, a sulfated polysaccharide negatively charged, dextran sulfate, was tested using a similar protocol. Dextran sulfate was selected since this molecule has a broad spectrum of antiviral activity against enveloped viruses such as herpes simplex virus (HSV), human cytomegalovirus, and vesicular stomatitis virus (Baba et al., 1988). Piret et al. (2000) demonstrated an inhibitor effect of dextran sulfate against various HSV-1 and HSV-2 strains by preventing binding of HSV to cell surface receptors and therefore their entry into cells. In the 1990s, several studies aiming to find new treatments to control Human Immunodeficiency Virus type 1 (HIV) infection showed that dextran sulfate interfered with membrane glycoprotein gp120 of HIV envelope and CD4 T lymphocytes (Bagasra and Lischner, 1988; Nakashima et al., 1989; Schols et al., 1990; Callahan et al., 1991). This effect was attributed to an inhibition of virus adsorption to host cell membrane (Baba et al., 1988). Due to potential inhibitor effects on enveloped viruses, this way was explored for OsHV-1 infection by combining *in vitro* and *in vivo* approaches.

## Materials and Methods

### Antiviral Antibody Production

Three OsHV-1 proteins encoded by ORFs 25, 41, and 72 and identified as putative membrane proteins by Davison et al. (2005), were selected for antiviral antibody production since they may correspond to structural proteins located on the surface of the viral envelope, and therefore play a key role in interaction between OsHV-1 and its host cells. This interaction especially occurs in the earliest stage of infection during attachment and entry of the virus into cells.

Polyclonal antibodies targeting proteins encoded by ORFs 25, 41, and 72 were produced by ProteoGenix (Schiltigheim, France) at 1 mg/mL. Briefly, the partial cDNA of each ORF was cloned in the pET-43.1a vector to express the protein with His tag in N-terminal position (cloning strategy: Ndel/XhoI). After purification of the three recombinant proteins, each one was then injected into rabbits. Polyclonal antibodies were separately purified using protein A affinity chromatography.

Polyclonal antibodies targeting proteins encoded by ORFs 25 and 72 were previously used by Martenot et al. (2016) to describe localization and tissue distribution of OsHV-1 proteins during an experimental OsHV-1 infection.

### Preparation of Oyster Tissue Homogenates

Tissue suspensions were prepared from experimentally infected or uninfected spat, according to the protocol developed by Schikorski et al. (2011a; 2011b). Two hundred microliters (μL) were sampled for DNA extraction and real time polymerase chain reaction (PCR) analysis to quantify viral DNA in tissue homogenates. Tissue homogenate positive and negative for viral DNA detection was called “viral suspension” and “control suspension,” respectively. A total of 100 μL was plated onto Zobell agar and incubated for 2 to 3 days at 22°C to control for the absence of bacteria in suspensions.

### Contact Between Preparation of Viral Suspension and Antiviral Antibodies or Dextran Sulfate

Viral suspension was directly used for *in vivo* and *in vitro* assays at 10^5^ viral DNA copies per μL or after an incubation period with antiviral antibodies or dextran sulfate before use.

Polyclonal antiviral antibodies (1 mg/mL) were incubated separately (30 μL of anti-ORF 25 or 30 μL of anti-ORF 41, or 30 μL of anti-ORF 72) or in combination (10 μL of anti-ORF 25, 10 μL of anti-ORF 41, and 10 μL of anti-ORF 72) with 1 mL of viral suspension.

A control was performed using a green fluorescent protein (GFP) Tag polyclonal antibody (A11122, Life Technologies) to determine the specificity of the effect induced by antiviral antibodies. As GFP antibody was concentrated at 2 mg/mL, 15 μL of this antibody were added to 1 mL of viral suspension.

A stock solution of dextran sulfate (1020-A, Euromedex) concentrated at 5 mg/mL was performed in artificial seawater (ASW) sterilized by autoclaving and filtered at 0.22 μM. This solution was then diluted in viral suspension to obtain a final concentration of 30 or 300 μg/mL.

All prepared suspensions with antiviral antibodies and dextran sulfate were incubated at 4°C overnight under gentle stirring.

### *In vivo* Trials: Biological Material and Experimental Infection

*Crassostrea gigas* spat produced in 2014 at the Ifremer hatchery located in Argenton (Brittany, France) and then reared at Ifremer facilities in Bouin (Vendée, France) were used for experimental infections by OsHV-1. The viral challenge was performed according to the protocol developed by Schikorski et al. (2011a, b). Briefly, spat were put in a myorelaxing solution containing magnesium chloride to open their valves before injecting suspension in the adductor muscle. Sixty spat (15 spat per tank) were used for each tested condition: viral suspension, viral suspension incubated with 30 μg/mL of dextran sulfate and viral suspension incubated with three anti-viral antibodies (in combination). Twenty spat distributed in 2 tanks (10 spat per tank) were used for each control: viral suspension incubated with anti-GFP antibodies (*n* = 20) (1), suspension prepared from uninfected spat incubated with dextran sulfate at 30 μg/mL (*n* = 20) (2), suspension prepared from uninfected spat incubated with antibody buffer (*n* = 20) (3), viral suspension incubated with the antibody buffer (*n* = 20) (4), and sterilized ASW (*n* = 20) (5). Control 1 aimed to validate the anti-viral specific effect in comparison to those obtained with anti-GFP antibodies. Controls 2, 3, and 4 aimed to ensure that dextran sulfate and antibody buffer did not induce oyster mortality or a degradation of viral particles, respectively. Control 5 was performed to ensure that spat mortality was specific to the injected suspension. After injection of suspensions in the adductor muscle, oysters were then placed in tanks containing 5 L of filtered seawater and kept in controlled conditions at 22°C without food supply.

Mortality was monitored three times per day during 1 week after intramuscular injection. Dead spat were collected and stored at −20°C for DNA extraction and OsHV-1 DNA quantification by real-time PCR.

### *In vitro* Trials

*In vitro* assays were performed by incubating hemolymph with viral suspension according to the protocol developed by Morga et al. (2017). For the present work, an important hemolymph volume was requisited and consequently hemolymph from *C. gigas* adults were used to realize *in vitro* trials. In this context, OsHV-1 replication was firstly investigated using hemolymph collected from six oyster families produced from two different genetic backgrounds (hereafter named from A to F). Four of them were unselected families (families A, B, C, and E) and the two others were selected for their higher resistance to OsHV-1 infection (families D and F). Oysters from families A to D shared the same genetic background whereas oysters from families E and F shared another genetic background. Their susceptibility to OsHV-1 infection was tested under experimental infection by cohabitation as well as in field condition (Table 1). Mortality rates were associated with high OsHV-1 DNA concentrations (data not shown). Due to the low amounts of oysters in families A, B, and C, and as they shared same genetic background and demonstrated a similar susceptibility to OsHV-1 infection in experimental challenges, hemolymph from these three family oysters was gathered to perform some *in vitro* trials.

**Table 1:** Mortality rates reported in the field and in experimental challenges with OsHV-1 for the six oyster families.

Oyster families	Mortality rate (field)	Mortality rate (experimental Challenge)
A	71%	86%
B	89%	85%
C	63%	85%
D	41%	31%
E	79%	98%
F	11%	11%

After cutting the edge of the oyster shell, hemolymph was collected from adductor muscle using a 1 ml syringe equipped with a needle (0.60 mm × 32 mm). Hemolymph collected from several oysters of a same family was pooled together and then filtered through a nylon grid of 70 μm (352350, Dutcher) to eliminate large particles. Hemolymph was held on ice before use to limit cell aggregation.

A total of 200 μL and 1.7 mL of hemolymph pool was sampled for OsHV-1 DNA and RNA detection, respectively.

For the *in vitro* assay, 1.7 mL of hemolymph, 119 μL of an antibiotic mix filtered at 0.22 μm (4 mg/mL of streptomycin, 11.6 mg/mL of penicillin, 5.1 mg/mL of neomycin, 3.3 mg/mL of erythromycin, 0.1 μL/mL of nystatin), and 850 μL of a viral suspension concentrated at 10^5^ viral DNA copies/μL were added in an Eppendorf 5 mL tube. A negative control was performed by replacing viral suspension with 850 μL of sterilized ASW. They were incubated at 19°C under gentle shaking during 0, 2, 4, 6, and 18 h. Four replicates were performed: two replicates for OsHV-1 DNA analysis and two replicates for OsHV-1 RNA analysis. After the incubation period, samples were centrifuged at 1,500 × g for 10 min at 4°C to obtain hemocyte pellets and the supernatant was removed. For viral DNA investigation, samples were stored at −20°C. For viral RNA analysis, 1 mL of TRIZOL^®^Reagent^TM^ () was added to the hemocyte pellet on ice and mixed fore few minutes, to be stored later at −80°C.

The effect of the three polyclonal antiviral antibodies and dextran sulfate was tested with the protocol described above. Hemolymph from adult oysters was added to a viral suspension incubated with antiviral antibodies or anti-GFP antibodies or dextran sulfate overnight at 4°C.

### Molecular Analysis

#### OsHV-1 DNA Detection

DNA was extracted from the mantle of dead spat sampled during OsHV-1 experimental infection (*in vivo* trials) with the QIAmp DNA mini Kit (Qiagen) according to the manufacturer’s instructions. DNA concentrations and quality were then measured using a NanoDrop spectrophotometer (Thermo Fisher Scientific). DNA was diluted at 5 ng/μL, and 5 μL of this solution was used to perform real time PCR analysis. The same DNA extraction protocol was used for hemocyte pellets collected during *in vitro* assays. Elution was performed in 50 μL of water for molecular biology, and 5 μL was directly used for real time PCR analysis.

As dextran sulfate caused PCR inhibition, another DNA extraction protocol was used for hemolymph incubated with this molecule. Magnetic beads of the NucliSENS^®^ easyMAG^®^ kit (Biomerieux, France) were used to capture DNA and eliminate PCR inhibitors. Elution was performed in 50 μL of elution buffer, and 5 μL was directly used for real time PCR analysis.

OsHV-1 DNA quantification was performed with real-time PCR based on TaqMan^®^ chemistry targeting a putative apoptosis inhibitor (Martenot et al., 2010). Briefly, 5 μL of DNA was added to the reaction mixture composed of 10 μL of Brilliant III Ultra-Fast QPCR Master Mix (Agilent Technologies), 0.4 μL of each primers (20 μM) OsHV1BF (forward) 5′-GTCGCATCTTTGGATTTAACAA-3′ and B4 (reverse) 5′-ACTGGGATCCGACTGACAAC-3′, 0.4 μL of TaqMan^®^ probe (10 μM) 5′-TGCCCCTGTCATCTTGAGGTATAGACAATC-3′, and 3.8 μL of distilled water. Amplification was performed in duplicate for each sample and accomplished using a Mx3000P real-time PCR thermocycler (Agilent). PCR conditions were 1 cycle at 95°C for 3 min, 40 cycles of amplification at 95°C for 10 s, and 60°C for 20 s. Assays included a standard curve and a negative control (5 μL of distilled water instead of the 5 μL of sample DNA). Results were expressed in viral DNA copies in one nanogram of total DNA for oyster tissue samples or in extracted DNA volume for hemocyte pellets.

#### Oyster and OsHV-1 RNA Quantification

Total RNA was extracted using the Ambion^®^ TRIZOL^®^ Reagent^TM^ (Life Technologies, Saint-Aubin, France) according to the manufacturer’s recommendations. RNA quality and amount were controlled with NanoDrop spectrophotometer (Thermo Fisher Scientific). A DNase treatment was performed with the Ambion^®^ TURBO DNA-free^TM^ (Life Technologies) according to the manufacturer’s instructions. A second RNA extraction was then realized using TRIZOL^®^ Reagent^TM^ to inhibit and eliminate the DNase.

A No RT (Reverse Transcription) was systematically performed after each DNAse treatment by real time PCR (SYBR^®^ Green chemistry) targeting the oyster elongation factor alpha (EF1 alpha) to control absence of oyster and virus genomic DNA. Five microliters of DNAse-treated RNA were added to the reaction mixture composed of 10 μL of Brilliant III ultra-Fast SYBR^®^ Green Master Mix 10X (Agilent Technologies), 2 μL of each primer concentrated at 1.5 μM, and 1 μL of distilled water. Amplification was performed in duplicate for each sample and accomplished using a Mx3000P real-time PCR thermocycler (Agilent). PCR conditions consisted of 1 cycle at 95°C for 3 min, 40 cycles of amplification at 95°C for 5 s, 60°C for 20 s, and followed by a dissociation stage (95°C for 1 min, 60°C for 30 s, and 95°c for 30 s).

After RNA quantification, first-strand cDNA synthesis was carried out using the Super-Script^®^ III First-Strand Synthesis System (Invitrogen) from 500 ng of DNAse-treated RNA. Due to inhibition problems with dextran sulfate, reverse transcription was conducted on 50 ng of DNAse-treated RNA for samples containing this molecule. A 1:30 dilution of the cDNA was realized and 5 μL was used for PCR investigation.

Three viral genes encoding a putative membrane protein (ORF 72), a putative dUTPase (ORF 75) and a putative apoptosis inhibitor (ORF 87) were targeted to evaluate viral transcript production. These genes were selected on the basis of results reported by Morga et al. (2017) and Segarra et al. (2014a, b).

Viral gene expression levels were calculated for each sample with the following formula: [1 / (Ct _viral ORF_ − Ct _EF_)] × 100 (Morga et al., 2017).

### Statistical Analysis

Data obtained from *in vivo* trials were analyzed with Generalized Linear Mixed Model (GLMM) to compare spat mortality between the different experimental conditions (Bolker et al., 2009).

Data obtained from *in vitro* trials were analyzed with the Shapiro–Wilk test and then a *t*-test (if the sample distribution was normal) or Mann–Whitney test (if normal distribution could not be demonstrated) by using XLSTAT-Pro^®^ 2014.5.03 software (Addinsoft, Paris, France). This statistical analysis was performed to compare viral transcript numbers and viral DNA amounts obtained from one *in vitro* condition test (virus incubated with antiviral antibodies or dextran sulfate) to those obtained from the control condition (virus alone) and for each targeting ORF. This approach was also used to compare viral transcript numbers and viral DNA amounts between adult oysters presenting a high susceptibility to OsHV-1 infection and adult oysters which are less susceptible to OsHV-1 infection.

## Results

### Effect of Antiviral Antibodies or Dextran Sulfate on OsHV-1 Experimental Infection (*in vivo* Trials)

**FIGURE 1:**
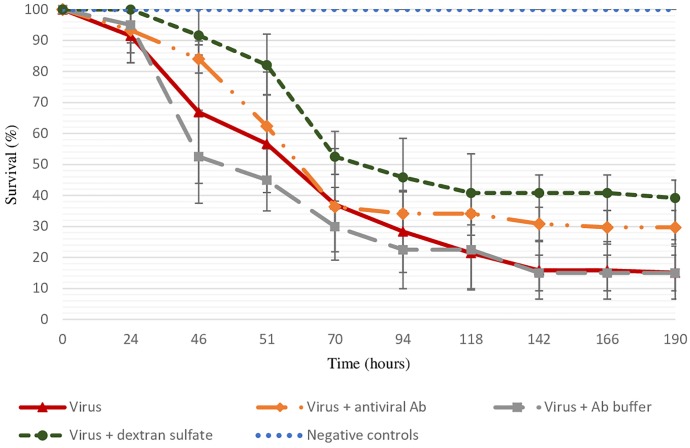
Survival rate of spat injected with viral suspension, viral suspension incubated with the three antiviral antibodies or with dextran sulfate (30 μg/mL), and negative controls (sterilized ASW, “control suspension” incubated with antibody buffer, “control suspension” incubated with dextran sulfate at 30 μg/mL) during 8 days. Error bars represent standard deviation of produced data.

As no spat mortality was recorded in different control conditions (sterilized ASW and antibodies buffer) and viral DNA copies per nanogram of total DNA in dead oysters were log 4.8 ± 0.8, mortality observed in tested conditions was considered to be due to viral infection (Figure 1). The final survival rate was significantly lower in positive control (viral suspension) in comparison to the virus incubated with the three antiviral antibodies (*p* = 0.0403) or with dextran sulfate (*p* = 0.0483) (Figure 1).

The specific effect of antiviral antibodies was investigated by a treatment of viral suspension with anti-GFP antibodies. Similar final mortality rates between positive control (70% ± 0%) and virus suspension incubated with anti-GFP antibodies (75% ± 7%) were reported.

### *In vitro* Protocol Validation With Hemolymph From Adult Oysters

#### OsHV-1 DNA Detection in Hemolymph Collected From Adult Oysters With Different Susceptibility to OsHV-1 Infection

**FIGURE 2:**
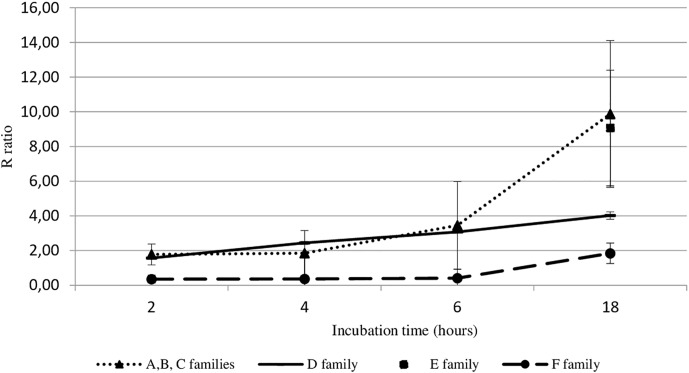
OsHV-1 DNA amount trend in haemocytes from different oyster families incubated with viral suspension through time (hours). The R ratio corresponds to the viral DNA amount at each time post-incubation in comparison with the viral DNA amount at 0 h post-incubation (T0) in haemolymph. Error bars represent standard deviation of produced data.

Viral DNA amount in hemolymph from adult oysters incubated with viral suspension was determined at each post-incubation time (2 h, 4 h, 6 h, or 18 h) and was then compared to viral DNA amount in hemolymph at 0 h post-incubation time (T0): R ratio (Figure 2). Precisely, the sample time T0 corresponds to the contact period between hemolymph and viral suspension during the centrifugation step that occurred just before the DNA extraction step. The higher the R ratio was, the higher the viral DNA amount was in the hemolymph. At 18 h post-incubation, the amount of OsHV-1 DNA was significantly higher in hemolymph from adult oysters presenting a high susceptibility to OsHV-1 infection (oysters from families A, B, C, and E) than those collected from adult oysters which are less susceptible to OsHV-1 infection (oysters from families D and F; *p* < 0.01) (Figure 2).

#### Number of Viral Transcripts in Hemolymph From Adult Oysters With Different Susceptibility to Oyster Infection at 18 h Post-incubation

**FIGURE 3:**
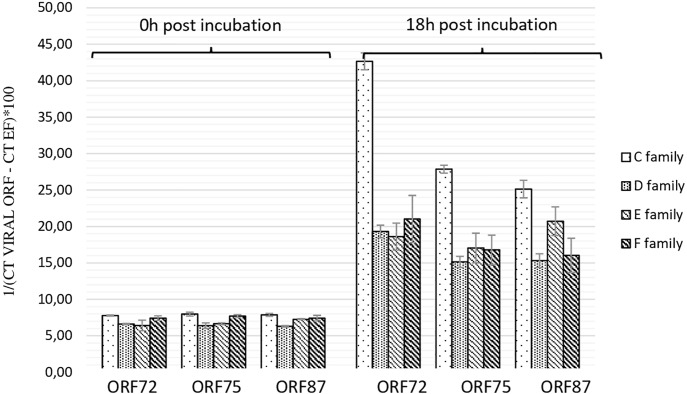
Number of viral transcripts in haemolymph from adult oysters presenting different susceptibility to oyster infection at 0 h and 18 h post-incubation for ORF 72, ORF 75, and ORF 87. Error bars represent standard deviation of produced data.

As the highest OsHV-1 DNA detection in hemolymph incubated with viral suspension was reported at 18 h post-incubation, this sample time was selected to evaluate the number of viral transcripts in the same *in vitro* conditions. The number of three viral transcripts (ORF 72, ORF 75, and ORF 87) was reported for hemolymph collected from adult oysters presenting different susceptibility to OsHV-1 infection, incubated with viral suspension (Figure 3). The mean transcript amount of the three targeted ORF at 18 h post-incubation was 2.95-fold higher than that at 0 h post-incubation, whatever the oyster susceptibility to OsHV-1 infection (*p* < 0.0001) (Figure 3). At 0 h post-incubation (T0) with viral suspension, no significant difference was observed between hemolymph collected from adult oysters presenting a high susceptibility to OsHV-1 infection (C and E oyster families) and those collected from adult oysters which are less susceptible to OsHV-1 infection (D and F oyster families; *p* > 0.05) (Figure 3). In contrast to 18 h post-incubation with viral suspension, ORF 87 transcript amount was significantly higher in hemolymph collected from adult oysters presenting a high susceptibility to OsHV-1 infection (oysters from families C and E) in comparison to viral transcript number in hemolymph collected from adult oysters which are less susceptibility to OsHV-1 infection (oysters from families D and F; *p* < 0.05) (Figure 3).

### Effect of Antiviral Antibodies on OsHV-1 Infection in *in vitro* Condition

#### Comparison of OsHV-1 DNA Amounts in Hemolymph Incubated With OsHV-1 Suspension Versus Hemolymph Incubated With OsHV-1 Suspension Containing Antiviral Antibodies

Viral DNA amount in hemolymph from adult oysters was determined at each time post-incubation (2 h, 4 h, and 6 h) for each condition: hemolymph incubated with viral suspension versus hemolymph incubated with viral suspension in presence of the three polyclonal antibodies. No significant difference of viral DNA copies per μL of DNA extract was observed between adult oyster hemolymph incubated with viral suspension (log 5.65 ± 0.06, log 5.62 ± 0.13, and log 5.89 ± 0.14) and viral suspension containing antiviral antibodies (log 5.68 ± 0.02, log 5.84 ± 0.11, and log 5.71 ± 0.09) at 1 h, 4 h, and 6 h post-incubation (the three *p*-values were superior to 0.05 for each post-incubation time). Viral DNA amounts obtained for adult hemolymph incubated with viral suspension containing anti-GFP antibodies were similar to those reported with hemolymph incubated with viral suspension at 6 h post-incubation (data not shown).

Secondly, a similar experiment was conducted by incubating viral suspension with polyclonal antibodies separately (Table 2) at 19°C for 6 h. Viral DNA amount was significantly lower in hemolymph incubated with viral suspension containing anti-ORF 25 antibodies (*p* = 0.011) or anti-ORF 41 antibodies (*p* = 0.013) in comparison with hemolymph incubated with viral suspension (Table 2). No significant difference of viral DNA amount was observed between hemolymph incubated with viral suspension containing anti-ORF 72 (*p* = 0.331) or anti-GFP (*p* = 0.619) antibodies and hemolymph incubated with viral suspension (Table 2).

**Table 2:** Number of viral DNA copies per μL of DNA extract (log10) in adult oyster haemolymph incubated with the viral suspension or with the viral suspension containing antiviral (anti-ORF 25, or anti-ORF 41, or anti-ORF 72) or anti-GFP antibodies at 6 h post-incubation (^∗^ for *p* < 0.05).

Conditions	TOh	T6 h	A = T6 h−T0 h
Virus	4.48	4.92	0.44
Virus + anti-ORF 25	4.51 ± 0.02	4.71 ± 0.01	0.20^∗^
Virus + anti-ORF 41	4.53 ± 0.04	4.67 ± 0.05	0.14^∗^
Virus + anti-ORF 72	4.36 ± 0.03	4.76 ± 0.02	0.40
Virus + anti-GFP	4.39 ± 0.12	4.86 ± 0.07	0.47

#### Number of Viral Transcripts in Hemolymph From Adult Oysters Incubated With OsHV-1 Suspension Versus Hemolymph Incubated With OsHV-1 Suspension Containing Antiviral Antibodies

**FIGURE 4:**
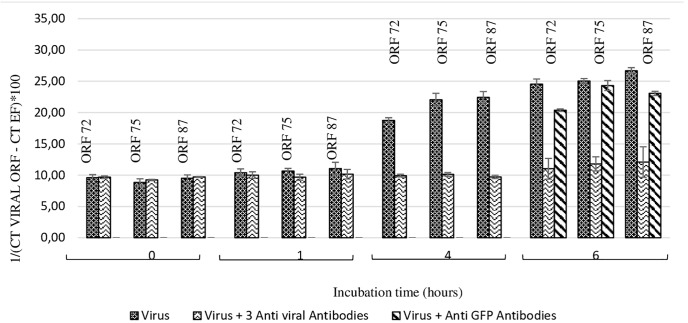
Number of viral transcripts in haemolymph from adult oysters incubated with the viral suspension or the viral suspension containing the three anti-viral or anti-GFP antibodies at 0 h, l h, 4 h, or 6 h post-incubation for ORF 72, ORF 75, and ORF 87. Error bars represent standard deviation of produced data.

At 4 h and 6 h post-incubation, the number of viral transcripts was significantly higher in hemolymph incubated with viral suspension in comparison with hemolymph incubated with OsHV-1 suspension containing the three antiviral antibodies (*p* values ranged from 0.002 to 0.014 for each of the three targeted ORFs and for both incubation periods) (Figure 4). No significant difference was reported between ORF75 transcript amounts in hemolymph incubated with OsHV-1 suspension and those incubated with viral suspension containing anti-GFP antibodies at 6 h post-incubation (*p* = 0.370) (Figure 4). There were significantly less ORF 72 (*p* = 0.016), ORF 75 (*p* = 0.006), and ORF 87 (*p* = 0.024) transcripts in hemolymph incubated with OsHV-1 suspension in the presence of antiviral antibodies than those incubated with viral suspension containing anti-GFP antibodies at 6 h post-incubation (Figure 4).

**FIGURE 5:**
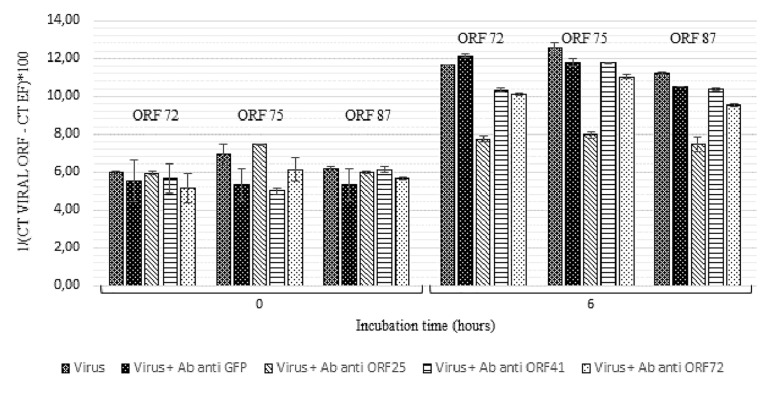
Number of viral transcripts in haemolymph from adult oysters incubated with the viral suspension or the viral suspension containing anti-viral or anti-GFP antibodies separately at 0 h and 6 h post-incubation for ORF 72, ORF 75, and ORF 87. Error bars represent standard deviation of produced data.

Secondly, a similar experiment was conducted by incubating viral suspension with polyclonal antibodies separately at 19°C for 6 h (Figure 5). Viral transcript amount was significantly lower in hemolymph incubated with viral suspension containing anti-ORF25, anti-ORF41 or anti-ORF72 antibodies in comparison with those incubated with viral suspension alone (*p* values were inferior to 0.016 for the three tested conditions and for each of the three targeted ORF, excepted for ORF 75 transcripts in the condition with viral suspension containing anti-ORF41 compared to viral suspension alone) (Figure 5). No significant difference was reported between ORF75 transcript amounts in hemolymph incubated with OsHV-1 suspension and those incubated with viral suspension containing anti-GFP antibodies at 6 h post-incubation (*p* = 0.076) (Figure 5). There were significantly less ORF 72, ORF 75, and ORF 87 transcripts in hemolymph incubated with OsHV-1 suspension in presence of anti-ORF25, anti-ORF41 or anti-ORF72 antibodies than those incubated with viral suspension containing anti-GFP antibodies at 6 h post-incubation (*p* values were lower than 0.028 for the three tested conditions and for each of the three targeted ORFs, with the exception of ORF 75 and ORF 87 transcripts in the condition with viral suspension containing anti-ORF41 compared to viral suspension containing anti-GFP antibodies) (Figure 5). No viral RNA was detected under the control condition (hemolymph alone).

### Effect of Dextran Sulfate on OsHV-1 Infection in *in vitro* Condition

#### Comparison of OsHV-1 DNA Amounts in Hemolymph Incubated With OsHV-1 Suspension Versus Hemolymph Incubated With OsHV-1 Suspension Containing Dextran Sulfate at 6 h Post-incubation

Viral DNA amount was significantly lower in hemolymph incubated with viral suspension containing dextran sulfate at 30 μg/mL (*p* = 0.016) or at 300 μg/mL (*p* = 0.007) in comparison with those incubated with viral suspension alone (Table 3).

#### Number of Viral Transcripts in Hemolymph From Adult Oysters Incubated With OsHV-1 Suspension Versus Hemolymph Incubated With OsHV-1 Suspension Containing Dextran Sulfate at 6 h Post-incubation

**FIGURE 6:**
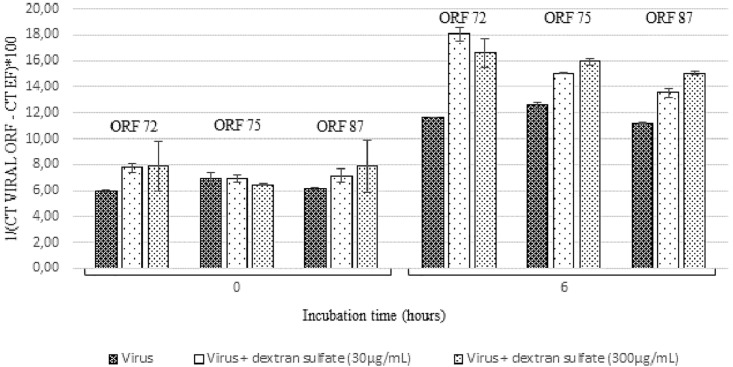
Number of viral transcripts in haemolymph from adult oysters incubated with the viral suspension or the viral suspension containing dextran sulfate at 30 μg/mL or 300 μg/mL at 0 h and 6 h post-incubation for ORF 72, ORF 75, and ORF 87. Error bars represent standard deviation of produced data.

At 6 h post-incubation, the amount of viral transcripts was significantly higher in hemolymph incubated with viral suspension containing dextran sulfate at 30 μg/mL (*p* values were inferior to 0.011 for each of the three targeted ORF) or 300 μg/mL (*p* values were inferior to 0.024 for each of the three targeted ORF) in comparison with those incubated with viral suspension alone (Figure 6). The number of viral transcripts was significantly lower in hemolymph incubated with viral suspension containing dextran sulfate at 30 μg/mL than in those incubated with viral suspension containing dextran sulfate at 300 μg/mL at 6 h post-incubation (*p* values were inferior to 0.031 for each of the three targeted ORFs, except for ORF 72 transcripts) (Figure 6). No viral RNA was detected under the control condition (hemolymph alone).

## Discussion

The infection process of herpesviruses begins with virus attachment to cells by numerous molecular bindings, followed by fusion of virion envelope with cell membrane, allowing viral capsid penetration in host cells. Viral entry mechanisms of herpesviruses constitutes a highly complex process which implicates several viral proteins and glycoproteins present in the virion lipid bilayer and different cellular receptors located on host cell surfaces (Spear and Longnecker, 2003; Spear, 2004; Agelidis and Shukla, 2015).

**Table 3:** Number of viral DNA copies per μL of DNA extract (log10) in adult oyster haemolymph incubated with the viral suspension or viral suspension containing dextran sulfate at 30 μg/mL or 300 μg/mL at 0 h and 6 h post-incubation (^∗^ for *p* < 0.05).

Conditions	TOh	T6 h	A = T6 h−T0 h
Virus	3.47	3.96	0.46
Virus + Dextran sulfate (30 μg/mL)	4.10 ± 0.02	4.15 ± 0.09	0.05^∗^
Virus + Dextran sulfate (300 μg/mL)	4.35 ± 0.34	4.18 ± 0.15	−0.17^∗^

To identify which OsHV-1 glycoproteins might be implicated in attachment of the virus to oyster cells, three viral proteins were first selected and polyclonal antibodies against these targets were used to try to inhibit interactions with cellular receptors, restricting entry of OsHV-1 into host cells. The selection of OsHV-1 putative membrane proteins was based on protein sequence homology and results obtained from transcriptomic and gene expression approaches during OsHV-1 experimental infection, which were conducted to better understand the OsHV-1 infection cycle (Davison et al., 2005; Jouaux et al., 2013; Rosani et al., 2014; Segarra et al., 2014b). Three proteins encoded by ORF 25, ORF 41, and ORF 72 appeared to be suitable candidates for the present investigation, since these proteins might correspond to proteins located on the viral envelope surface, and therefore play a key role in interactions between virus and host. However, they do not share homology with already known membrane glycoproteins from other herpesviruses as all other OsHV-1 putative membrane proteins.

Results from experimental trials showed a significant lower mortality rate of spat injected with viral suspension incubated in presence of the three antiviral antibodies in comparison with spat injected with viral suspension alone (70 and 85%, respectively). These results must be considered in these controlled conditions, antiviral antibody concentrations, and genetic background of oysters. The mortality rate recorded during *in vivo* trials was associated with OsHV-1 infection, since higher viral DNA amounts were detected in dead oysters, which were in accordance with those previously reported (Oden et al., 2011; Schikorski et al., 2011a,b). The impact of chemical composition of antibody buffer was controlled by injecting buffer solution in the adductor muscle of spat and no mortality was recorded. The specific effect of antiviral antibodies was investigated by injecting into the spat adductor muscle a viral suspension incubated with anti-GFP antibodies. As no significant difference of mortality rate was reported between oysters injected with viral suspension incubated with anti-GFP antibodies and viral suspension alone, the results suggest that the observed effect in the presence of the three antiviral antibodies was specific to the target of antiviral antibodies. Even if this experimental approach presents the advantage of injecting the same concentration of viral suspension and antibodies into each oyster, injection into the adductor muscle did not constitute the natural way for OsHV-1 infection and could explain the low difference of mortality rates between oysters injected with viral suspension incubated with antiviral antibodies and those injected with the viral suspension alone. No information is available on antibody stability after they have been injected into oysters either. Other experimental approaches, consisting of putting uninfected oysters into contact with experimentally infected ones, were not chosen for the present study since the required antibody amounts were too high. In this context *in vitro* approaches were implemented from a protocol developed by Morga et al. (2017) using spat hemolymph to better control ligand/cellular receptor interactions and to limit dilution effects that occurred in *in vivo* trials. This protocol was firstly adapted to hemolymph collected from adult oysters to sample larger amounts of circulating cells. Previous works reported OsHV-1 DNA, RNA, and protein detection in adult oysters, suggesting that OsHV-1 might enter in adult hemolymph/hemocytes (Arzul et al., 2002; Barbosa-Solomieu et al., 2005; Segarra et al., 2014a; Azéma et al., 2016). Additionally, oysters selected for their higher susceptibility to OsHV-1 show high mortality related to OsHV-1 infection at all life stages, including the adult stage (Azéma et al., 2017).

**Table 4:** Trends of viral DNA and RNA detection in adult oyster haemolymph incubated with OsHV-1 suspension in presence of anti-viral (anti-ORF25, anti-ORF41, and anti-ORF72; separately or in mixture).

	Three antiviral antibodies	Anti-ORF25	Anti-ORF41	Anti-ORF72
OsHV-1 DNA	–	↘	↘	–
ORF 72 transcripts	↘	↘	↘	↘
ORF 75 transcripts	↘	↘	–	↘
ORF 87 transcripts	↘	↘	↘	↘

*Arrows correspond to significant decrease of OsHV-1 DNA amount or viral transcript number in haemolymph incubated with the viral suspension in presence of antibodies in comparison with those incubated with the viral suspension alone. A dash was indicated no significant difference between the two incubation conditions (viral suspension incubated in presence or without antibodies).*

OsHV-1 RNA and DNA detection in adult hemolymph incubated with OsHV-1 suspension showed that OsHV-1 has the ability to recognize cellular receptors located on hemocyte’s surface and to enter in these immune cells, regardless of the hemolymph origin (spat or adult oyster) and susceptibility of oysters to OsHV-1 infection (Figure 3). This contrast with the absence of viral RNA detection 144 h post-injection into the adductor muscle of adult oysters suggests that adult oysters might be able to control viral replication (Segarra et al., 2014a). A significant higher number of viral transcripts in adult oysters showing high susceptibility to OsHV-1 infection at 18 h post-incubation is in agreement with a genetic basis of resistance to viral infection as previously reported (Dégremont et al., 2015; Azéma et al., 2017). The present results are also in accordance with those obtained by Segarra et al. (2014b), who investigated OsHV-1 infection in spat presenting high and low susceptibility level to OsHV-1 infection and reported a significant difference of OsHV-1 DNA and RNA amounts between different oyster families in association with dissimilar host gene expression profiles. In light of these results, hemolymph from adult oysters presenting a high susceptibility to OsHV-1 infection was selected to test for the antiviral antibody effect on OsHV-1 in *in vitro* conditions. At 6 h post-incubation, a significant difference in the number of viral transcripts was observed in hemolymph incubated with viral suspension containing three viral antibodies or antiviral antibodies targeting proteins encoded by ORF 25, ORF 41, and ORF 72 in comparison with hemolymph incubated with viral suspension alone (Table 4). These results lead to the assumption that the three targeted viral proteins, and mainly protein encoded by ORF 25, might be implicated in virus attachment to oyster cells. However, the detection of viral DNA or RNA in hemolymph incubated with the viral suspension incubated with antiviral antibodies (either separately or in mixture) suggested that other OsHV-1 proteins were required to allow virus attachment to cellular receptors or/and that antiviral antibody concentrations were not sufficient to completely inhibit interactions between ligands and cellular receptors. It is highly probable that other OsHV-1 proteins are implicated in this stage since the attachment of herpes simplex virus 1 (HSV-1) and herpes simplex virus 2 (HSV-2) required at least four viral envelop glycoproteins (gB, gD, gH, and gL), which are absolutely essential for HSV-1 and HSV-2 entry into host cells (Agelidis and Shukla, 2015). Some of them bind to cellular receptors, increasing virion number around cells, whereas other glycoproteins recognize specifically cellular receptors for membrane fusion allowing virions to enter cells (Spear and Longnecker, 2003). Further investigations need to be conducted to better characterize molecular interactions at the ligand/receptor level and identify other viral glycoproteins which are implicated in virus attachment to oyster cells. In addition, molecular interactions between OsHV-1 proteins and antiviral antibodies can be reversible (no destructive effect) and might be unstable in the experimental conditions tested. Consequently, OsHV-1 can finally achieve entry into host cells and begin its replication cycle until induced spat death in *in vivo* trials. Different effects observed between three antiviral antibodies against ORF 25, ORF 41, and ORF 72 proteins could be due to their specific role in virus attachment to cells or the number of these proteins on the OsHV-1 surface.

Herpes simplex virus adsorption to host cells is mediated by several cellular molecules, which act as cofactors, including herpesvirus entry mediator (HVEM), nectin-1, and glycosaminoglycans (Whitbeck et al., 1997; Connolly et al., 2001; Tiwari et al., 2006; Farooq et al., 2010; Karasneh and Shukla, 2011). Among glycosaminoglycans, heparan sulfate proteoglycans (HSPG) and chondroitin sulfate, which are present on the cell surface, were reported to be involved in the step of viral entry (Dyer et al., 1997; Spear and Longnecker, 2003; Agelidis and Shukla, 2015). Dextran sulfate, a negatively charged sulfated polysaccharide, was used as a glycosaminoglycan analog, and two concentrations of this molecule were tested in the present study to explore the nature of virus/host interactions (Dyer et al., 1997). Mortality was significantly lower for oysters injected with dextran sulfate added in viral suspension in comparison with those injected with viral suspension alone (60 and 85%, respectively). *In vitro* trials revealed that OsHV-1 DNA amounts in hemolymph incubated with viral suspension in the presence of dextran sulfate were significantly lower than those detected in hemolymph incubated with viral suspension alone. On the contrary, the number of viral transcripts was higher in hemolymph incubated with OsHV-1 suspension in the presence of dextran sulfate than in hemolymph incubated with viral suspension alone. Taken together, these results suggest that dextran sulfate might play an antiherpetic effect (decrease of final mortality rates and viral DNA production in *in vitro* trials) but does not affect viral transcription. A hypothetic effect of dextran sulfate on OsHV-1 capsid integrity was not demonstrated in the present study and does not account for decrease of final mortality rates or viral DNA production in *in vitro* trials. The effect of dextran sulfate differs in respect with studied viruses even if dextran sulfate is mainly known to inhibit viral attachment to target cells (Vaheri, 2007). However, Dyer et al. (1997) reported that dextran sulfate added either prior to or during inoculation stimulated herpes simplex virus 1 (HSV-1) but not herpes simplex virus 2 (HSV-2) infection and occurred during an early, energy-independent step in infection. Glycosaminoglycans in HSV infection constitute an efficient matrix for virus adsorption. On the contrary Huemer et al. (1992) showed that dextran sulfate inhibited binding of HSV glycoprotein C to complement third component (C3b). Inhibition effects change in respect to dextran sulfate concentration and molecular weight dextran sulfate (Huemer et al., 1992). In addition, *in vitro* experiments revealed that dextran sulfate significantly reduced the number of lytic plaques caused by pseudorabies virus (Suid herpesvirus 1), but did not modify the virulence of this virus in mice (Ramos-Kuri et al., 1990). A similar trend of the dextran sulfate effect was reported for influenza A virus strain at the initial and late stages of the viral infection process, which might suppress its replication (Yamada et al., 2012, 2014). More precisely, dextran sulfate is associated with inhibition of hemagglutinin-dependent fusion activity (initial stage of viral infection process) and may also be involved in inhibition of neuraminidase activity by dextran sulfate negative charge (later stage of viral infection) (Yamada et al., 2012, 2014). The potential inhibitor effect of dextran sulfate on Sendai virus fusion and erythrocyte ghosts was also described where dextran sulfate likely interacted preferentially with Sendai virion surfaces (Ohki et al., 1991). Supplementary investigation was required to reinforce these results by studying a dose-dependent effect of dextran sulfate. In addition, the antiherpetic effect of other polyanions such as heparin and chondroitin sulfate could be tested to complete the present results.

## Conclusion

Results obtained from assays conducted on hemolymph from adult oysters presenting different levels of susceptibility to OsHV-1 infection revealed that the virus is able to enter immune cells, product DNA and RNA regardless of the stage of oyster development or the genetic background.

Original and nondestructive approaches were applied in the present study to identify OsHV-1 proteins which might be implicated in virus attachment on host cells. This is the first time that one OsHV-1 putative viral envelop protein encoded by ORF 25 is suggested to be a potential candidate for involvement in the initial stage of the viral infection process and subsequently these results contribute to a better understanding of viral infection pathogenesis.

The antiherpetic effect of dextran sulfate was investigated against OsHV-1 infection and revealed an interesting approach to limit viral infection in controlled conditions such as oyster hatcheries or nurseries, and it therefore needs to be explored and tested more in combination with other polysaccharides.

## Data Availability

The raw data supporting the conclusions of this manuscript will be made available by the authors, without undue reservation, to any qualified researcher.

## Author Contributions

CM and TR conceived the experimentations. CM and NF realized the experimentations. LD provided the oysters. J-BL realized a part of the statistical analysis. CM and TR wrote the first draft of the manuscript. All authors reviewed the manuscript and approved the version to be submitted for publication.

## Conflict of Interest Statement

The authors declare that the research was conducted in the absence of any commercial or financial relationships that could be construed as a potential conflict of interest.
